# Terminalia Chebula Extract Replacing Zinc Oxide Enhances Antioxidant and Anti-Inflammatory Capabilities, Improves Growth Performance, and Promotes Intestinal Health in Weaned Piglets

**DOI:** 10.3390/antiox13091087

**Published:** 2024-09-05

**Authors:** Tao Wang, Yuying Li, Lichen Yin, Jiashun Chen, Pengjun Shi, Fang Wang, Kangle Wu, Kang Yao, Yulong Yin

**Affiliations:** 1 Laboratory of Animal Nutritional Physiology and Metabolic Process, Key Laboratory of Agro-Ecological Processes in Subtropical Region, National Engineering Laboratory for Pollution Control and Waste Utilization in Livestock and Poultry Production, Institute of Subtropical Agriculture, Chinese Academy of Sciences, Changsha 410125, China; 2 University of Chinese Academy of Sciences, Beijing 100008, China; 3 Hunan Provincial Key Laboratory of the Traditional Chinese Medicine Agricultural Biogenomics, Institute of Bast Fiber Crops, Chinese Academy of Agricultural Sciences, Changsha 410205, China; 4 Animal Nutritional Genome and Germplasm Innovation Research Center, College of Animal Science and Technology, Hunan Agricultural University, Changsha 410128, Chinajschen@hunau.edu.cn (J.C.)

**Keywords:** terminalia chebula extract, immune function, antioxidant capacity, intestinal health, piglets

## Abstract

This study aimed to assess the effects of substituting zinc oxide with terminalia chebula extract (TCE) on growth performance, antioxidant capacity, immune function, and intestinal health in weaned pigs. Initially, 72 weaned Duroc × Landrace × Large White piglets, 28 days old with an initial weight of 7.43 ± 0.14 kg, equally divided by gender, were randomly assigned into three groups, with six replicates and four piglets per replicate. They were fed a basal diet (CON group), a diet containing 2 g/kg zinc oxide (ZnO group), or 2 g/kg TCE (TCE group) for a duration of 28 days. Subsequently, to further confirm the most appropriate levels of TCE in piglets, 96 piglets of the same breeds and age, with an initial weight of 7.42 ± 0.12 kg, also equally divided by gender, were randomly assigned into four groups, each with six replicates and four piglets per replicate, and fed a basal diet (CON group), or diets supplemented with 1 g/kg TCE (LTCE group), 2 g/kg TCE (MTCE group), or 4 g/kg TCE (HTCE group) for a duration of 28 days. The results demonstrated that both TCE and ZnO reduced diarrhea rates (*p* = 0.001) and enhanced average daily gain (ADG) (*p* = 0.014) compared to the control group. TCE at 1 g/kg and 4 g/kg reduced the feed to gain ratio (*p* = 0.050). Dietary supplementing with TCE and ZnO increased serum total antioxidant capacity (T-AOC) (*p* = 0.020). Various doses of TCE also increased jejunal IgA (*p* = 0.000) levels and IL-10 expression (*p* = 0.004), and decreased the levels of TNF-α in both serum (*p* = 0.043) and jejunal mucosa (*p* = 0.000). Notably, TCE reduced the crypt depth (CD) of the duodenal (*p* = 0.007) and increased the villus height (VH) of the ileal (*p* = 0.045), and with increased dosage, there was a rise in the villus height to crypt depth ratio (VH:CD) in the duodenum (*p* = 0.000) and jejunum (*p* = 0.001). Higher abundances of *Lactobacillaceae* (*p* = 0.000) and lower levels of *Streptococcaceae* (*p* = 0.000) and *Peptostreptococcaceae* (*p* = 0.035) in cecal contents were fed the ZnO and TCE pigs compared with CON pigs. Therefore, TCE was firstly presented as being able to replace zinc oxide, improve intestinal morphology, and enhance antioxidant and immune functions, thus safeguarding intestinal mucosal health and promoting piglet growth.

## 1. Introduction

During weaning, piglets face physiological, environmental, and dietary changes that diminish their antioxidant and anti-inflammatory capacities, impacting intestinal health and leading to diarrhea, growth retardation, and even death [1,2,3]. Zinc oxide (ZnO) has been shown to enhance growth performance and effectively reduce diarrhea in piglets, thereby improving production performance [4]. However, the use of ZnO in feeds poses environmental pollution risks, affects the absorption of other nutrients by piglets, and contributes to the emergence of resistance for pathogenic bacteria [5]. Our previous studies have indicated that amino acids and plant extracts can improve intestinal health and alleviate diarrhea in weaned piglets, serving as alternatives to ZnO [6,7,8]. Among these alternatives, natural plant extracts are gaining widespread attention for their potential to inhibit harmful bacteria, enhance antioxidant and immune responses, and are considered environmentally friendly with broad application prospects [9].

Terminalia chebula (TC, Combretaceae), commonly known as black myrobalan or ink tree, is a deciduous tree from the Combretaceae family, widely distributed in tropical and subtropical regions around the world. It is extensively used in traditional Chinese and Tibetan medicine for its antioxidant, anti-inflammatory, and gastroprotective effects [10,11,12]. Extracts from TC (TCE) have been identified as potential feed additives that can enhance intestinal health in piglets. In addition, TCE can reduce the harmful bacteria in the animal gut while increasing beneficial bacteria such as *Lactobacilli*, thus enhancing nutrient digestion, alleviating weaning stress, and improving productivity and performance [13]. TCE also improve antioxidant and anti-inflammatory capacities in weaned bull calves [13]. Our previous research has shown that 600 mg/kg TCE can enhance immune function and antioxidant capacity in yellow broilers [14]. The major bioactive components of TCE include tannins (hydrolyzable tannins), triterpenoids, berberine, phenolic acids, and flavonoids [11,12]. Among them, tannins and berberine possess astringent, antimicrobial, anti-inflammatory, and antioxidant properties and can improve intestinal health and growth performance [15]. Although TCE has been used to some extent in animal production, studies on its effectiveness in substituting ZnO in piglet diets are still insufficient. In addition, the mechanisms by which TCE improves growth performance and intestinal health in weaned piglets require further investigation. Thus, this study aims to evaluate how TCE can substitute ZnO by enhancing antioxidant capacity, modulating inflammatory markers, improving intestinal barrier function, and optimizing gut microbiota to enhance growth performance and reduce diarrhea in piglets, providing a theoretical basis for the inclusion of TCE in piglet diets.

## 2. Materials and Methods

The procedures in this study were agreed to by the Animal Care and Use Committee of the Institute of Subtropical Agriculture, Chinese Academy of Science (no. ISA-2018-4-25).

### 2.1. Terminalia Chebula Extract

The TCE (powder form) was provided by Wuxi Zhengda Biotechnology Co., Ltd. (Wuxi, China), with active ingredients including chebulic tannins (45.0%), polysaccharides (14.2%), and flavone (3.1%).

### 2.2. Experimental Animals, Design, and Diets

Seventy-two weaned piglets (Duroc × Landrace × Large White, 28 days old, half male and half female, initial weight 7.43 ± 0.14 kg), equally divided by gender, were randomly assigned to three groups with six replicates per group and four piglets per replicate. They were fed a basal diet (CON), or the CON diet supplemented with either 2 g/kg ZnO or 2 g/kg TCE. The doses of TCE (2 g/kg) were selected according to the recommendation of a feed-additive company and preliminary feeding experiment (unpublished data). In order to further confirm the most appropriate levels of TCE in piglets, a supplementary experiment was performed. Specifically, ninety-six weaned piglets (Duroc × Landrace × Large White, 28 days old, initial weight 7.42 ± 0.12 kg), also equally divided by gender, were randomly divided into four groups: a control group fed a basal diet (CON), a low-dose TCE group (LTCE) with 1 g/kg TCE added to the basal diet, a medium-dose TCE group (MTC) with 2 g/kg TCE, and a high-dose TCE group (HTCE) with 4 g/kg TCE. The basal diet was formulated according to the recommendations of the National Research Council (NRC, 2012) (Table 1). Both trials lasted for 28 days with ad libitum feeding, and the pigs of each replicate were raised together. Feed was weighted before adding into trough, and the residual feed was collected and weighted in the next morning to calculate the feed consumption. At 0 and 28 days, all piglets were weighed to record, and the average daily gain (ADG) was analyzed according to the weights of per replicate. The average daily feed intake (ADFI) was calculated based on the consumption of feed every day from each replicate. ADG, ADFI, and feed-to-gain ratio (F:G) of piglets in each group were calculated on the basis of each replicate, the results were presented as the average value per replicate. Diarrhea rates were monitored according to the methods recommended by Wang et al. (2021) [16].

### 2.3. Sample Collection

On days 28 of both experiments, after fasting and weighing, one male piglet from each replicate (n = 6) close to the average weight was selected. Before slaughter, blood samples (5 mL) from the anterior vena cava were collected in blood-collecting vessel without anticoagulant, allowed to settle for 30 min, then centrifuged at 1500× *g* for 10 min to prepare serum, which was stored at −20 °C for the measurements of antioxidant and immune indices. During slaughter, the intestinal segments were quickly removed and separated. Mucosal samples from the jejunum were carefully scraped with sterile glass slides, rapidly collected, flash-frozen in liquid nitrogen, and stored at −80 °C for the analysis of antioxidant and immune indices. About 3 cm long segments from the middle of the duodenum, jejunum, and ileum were fixed in 10% neutral buffered formalin for histological examination of intestinal tissues. In pigs from the replacing ZnO with TCE trials, the ceca were ligated, wrapped in tin foil, and stored in liquid nitrogen for microbial analysis.

### 2.4. Diarrhea Incidence

During the formal experiment, the fecal contamination in the anus of each piglet was carefully observed during feeding every day, and the first diarrhea and diarrhea of each repetition were recorded in detail. The diarrhea incidence and fecal consistency scores (0 = normal feces, 1 = soft feces, 2 = mild diarrhea, 3 = severe diarrhea) were determined by a trained investigator with no prior knowledge of the dietary treatment assignment. Diarrhea rate (%) was calculated as the number of pigs with diarrhea × the number of days with diarrhea/(the total number of pigs × the number of study days).

### 2.5. Serum and Jejunal Mucosal Antioxidant and Immune Indices

Jejunal mucosal samples were homogenized in cold physiological saline (0.9%) at a ratio of 1:9 (weight (g) to volume (mL)), then centrifuged at 3000× *g* for 15 min at 4 °C. The total antioxidant capacity (T-AOC), catalase (CAT), glutathione peroxidase (GSH-Px), superoxide dismutase (SOD) activity, and malondialdehyde (MDA) levels in serum and jejunal mucosa, were measured using kits provided by Nanjing Jiancheng Bioengineering Institute (Nanjing, China). Serum and jejunal mucosal levels of tumor necrosis factor-alpha (TNF-α), interferon-alpha (IFN-α), immunoglobulins (IgA, IgG, IgM), and cytokines (IL-1β, IL-6, IL-10) were assessed using pig-specific enzyme-linked immunosorbent assay (ELISA) kits from Jiangsu Meimian Industrial Co., Ltd. (Yancheng, China).

### 2.6. Intestinal Morphology

Tissue samples were processed through washing, dehydration, clearing, paraffin embedding, and then sectioned using a LEICA RM2135 microtome. The sections were further processed through spreading, staining, and cover-slipping. The average villus height (VH), crypt depth (CD), and the ratio of villus height to crypt depth (VH:CD) for each sample were observed and measured using Caseviewer software 2.3. One slide per animal was measured, and five well-oriented villi of each pig at five different views were used to determine these indices.

### 2.7. Cecal Microbiota Composition

The total bacterial genomic DNA from microbial community of digesta samples in caecal was performed using a DNA Kit (Qiagen, Hilden, Germany) according to the manufacturer’s methods. Briefly, the integrity of the DNA was determined on 1% (weight (g) to volume (mL)) agarose gels, and the yield and purity of DNA were checked. The 16S rRNA of the caecal digesta samples was amplified with the V3-V4 region (338F ACTCCTACGGGAGGCAGCAG and 806R GGACTACHVGGGTWTCTAAT). Purification of PCR product was performed by AxyPrep DNA Gel Extraction Kit (Axygen Biosciences, Union City, CA, USA). Amplicon libraries were sequenced based on Illumina HiSeq PE250 platform (Illumina, San Diego, CA, USA) for paired-end reads of 300 bp. After discarding the low-quality sequencing reads, operational taxonomic unit (OTU) clustering was conducted and delimited at the cutoff of 97% by Uparse software version 7.1. The taxonomy of each OTU representative sequence was analyzed by RDP Classifier version 2.2 against the 16S rRNA database (Silva v138) using a confidence threshold of 70% [16].

### 2.8. RT-PCR

Jejunal mucosal samples were homogenized in 1 mL Trizol reagent (TaKaRa, Dalian, China), and total RNA was extracted according to the manufacturer’s instructions. RNA purity was assessed by spectrophotometry (Beckman Coulter DU800; Beckman Coulter Inc., Brea, CA, USA) at 260 and 280 nm, with OD260: OD280 ratios between 1.8 and 2.0. cDNA was synthesized using the PrimeScript RT reagent kit with gDNA Eraser (TaKaRa, Dalian, China) as per the manufacturer’s protocol. Real-time quantitative PCR was conducted on a CFX96 Real-Time System (Bio-Rad Laboratories, Inc., Hercules, CA, USA) to measure the expression levels of TNF-α, IFN-γ, IFNα1, IFNβ1, IL-1β, IL-4, IL-6, and IL-10, with β-actin serving as the internal reference transcript. Specific primers (sequences in Table 2) were synthesized by Sangon Biotech (Shanghai, China).

### 2.9. Statistical Analysis

All data are presented as mean (the group averages) ± standard error of the mean (SEM). Statistical analysis was performed using one-way ANOVA followed by Tukey’s test (IBM SPSS Statistics software 20). For the microbiota data, the abundance of microbiota composition was assessed by the Kruskal–Wallis test. A significance level of *p* < 0.05 was used. Differences were considered statistically different at *p* < 0.05. All figures were generated using GraphPad Prism 9.

## 3. Results

### 3.1. Effects of Replacing ZnO with TCE on Piglet Growth Performance, Diarrhea Rate, and Serum and Intestinal Mucosal Antioxidant Indices

**Figure 1:**
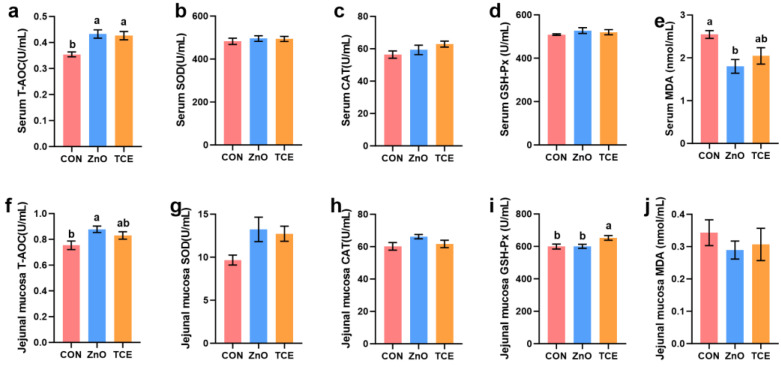
Effects of replacing ZnO with TCE on piglet serum and intestinal mucosal antioxidant indices (n = 6). (**a**) Serum T-AOC; (**b**) Serum SOD; (**c**) Serum CAT; (**d**) Serum GSH-px; (**e**) Serum MDA; (**f**) Jejunal mucosa T-AOC; (**g**) Jejunal mucosa SOD; (**h**) Jejunal mucosa CAT; (**i**) Jejunal mucosa GSH-px; (**j**) Jejunal mucosa MDA. Bars sharing different letter notations (a, b) are significantly different from each other (*p* < 0.05).

As shown in Table 3, compared to the CON group, both ZnO and TCE groups increased the ADG (*p* = 0.014) and reduced the diarrhea rate in piglets (*p* = 0.002). Although there was no difference in F:G, ZnO and TCE showed a trend towards reduction (*p* = 0.097). Dietary supplementation of ZnO and TCE increased serum T-AOC levels (Figure 1, *p* = 0.002). ZnO reduced serum MDA (*p* = 0.011) levels and increased jejunal mucosal T-AOC levels (*p* = 0.029). Notably, TCE enhanced jejunal mucosal GSH-Px levels compared with CON and ZnO (*p* = 0.026).

### 3.2. Effects of Replacing ZnO with TCE on Piglet Immunity

**Figure 2:**
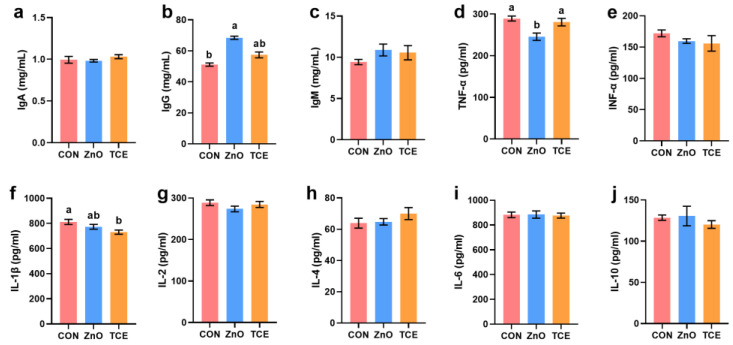
Effects of replacing ZnO with TCE on serum inflammatory cytokines (n = 6). (**a**) IgA; (**b**) IgG; (**c**) IgM; (**d**) TNF-α; (**e**) INF-α; (**f**) IL-1β; (**g**) IL-2; (**h**) IL-4; (**i**) IL-6; (**j**) IL-10. Bars sharing different letter notations (a, b) are significantly different from each other (*p* < 0.05).

**Figure 3:**
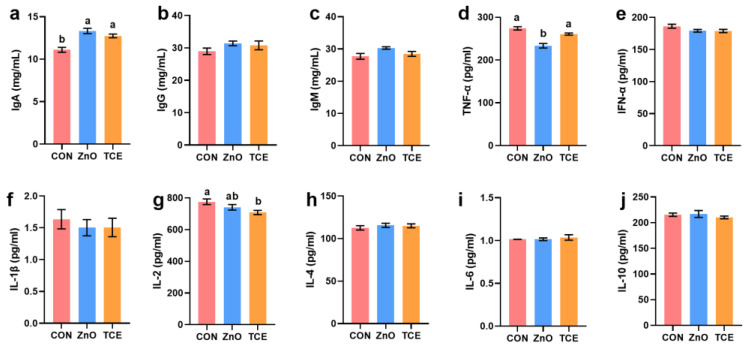
Effects of replacing ZnO with TCE on piglet jejunal mucosa inflammatory cytokines (n = 6). (**a**) IgA; (**b**) IgG; (**c**) IgM; (**d**) TNF-α; (**e**) IFN-α; (**f**) IL-1β; (**g**) IL-2; (**h**) IL-4; (**i**) IL-6; (**j**) IL-10. Bars sharing different letter notations (a, b) are significantly different from each other (*p* < 0.05).

**Figure 4:**
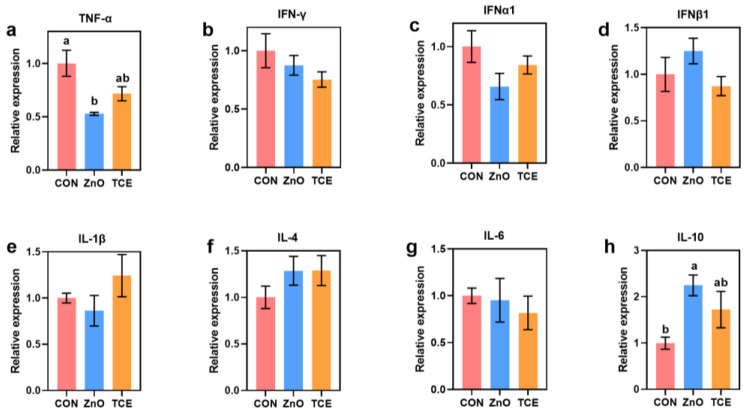
Effects of replacing ZnO with TCE on piglets relative to expression of inflammatory cytokines in the jejunal mucosa (n = 6). (**a**) TNF-α; (**b**) INF-γ; (**c**) IFN-α1; (**d**) IFN-β1; (**e**) IL-1β; (**f**) IL-4; (**g**) IL-6; (**h**) IL-10. Bars sharing different letter notations (a, b) are significantly different from each other (*p* < 0.05).

Compared to the control group, ZnO increased serum IgG levels (*p* = 0.047) and decreased TNF-α levels (*p* = 0.043), while there were no significant changes in the TCE group. However, TCE reduced serum IL-1β levels (*p* = 0.003, Figure 2). Additionally, TCE and ZnO also increased jejunal mucosal IgA (*p* = 0.000) levels, while TCE decreased IL-2 (*p* = 0.031) levels (Figure 3). Analysis of gene expression related to inflammatory cytokines revealed that ZnO decreased TNF-α expression (*p* = 0.006) and increased IL-10 expression (*p* = 0.018), while TCE showed no significant differences from the control group (*p* > 0.05, Figure 4).

### 3.3. Effects of Replacing ZnO with TCE on Piglet Intestinal Morphology

**Figure 5:**
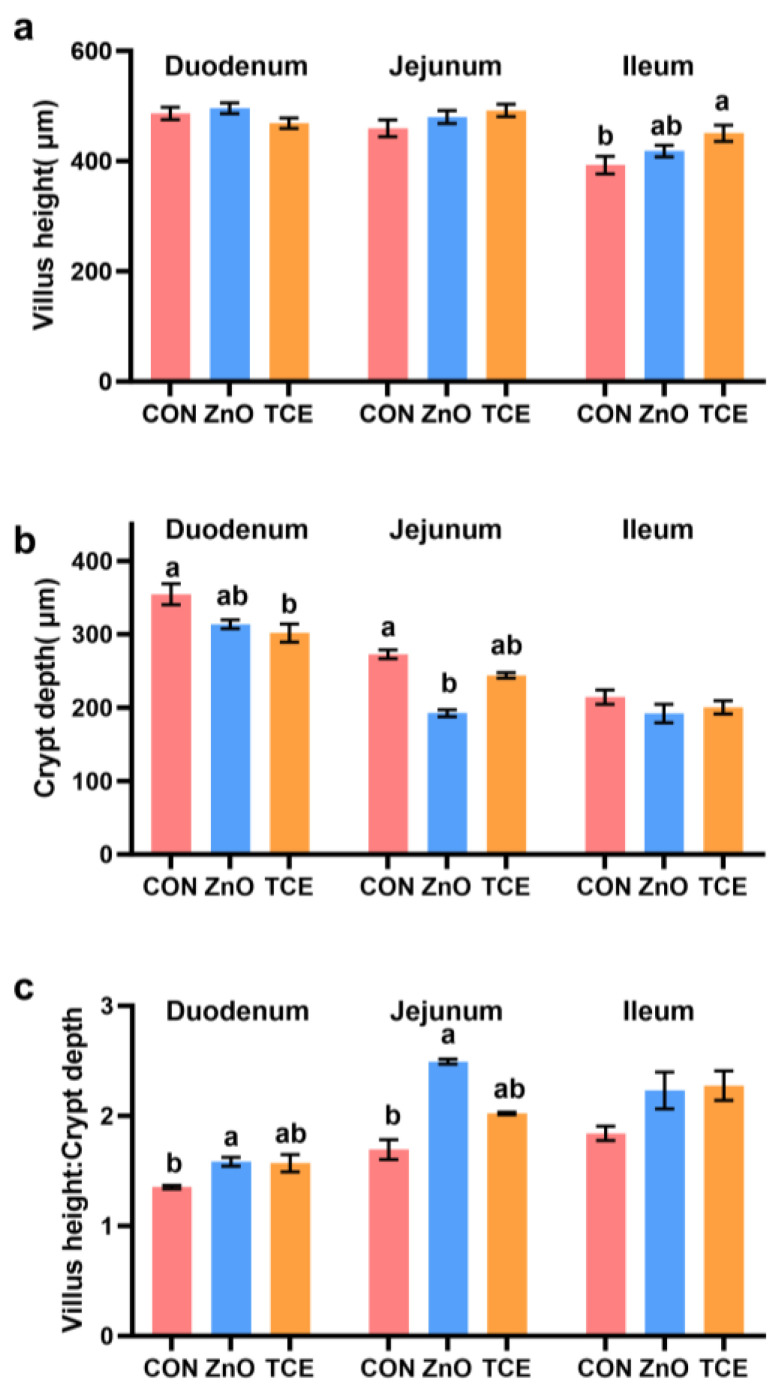
Effects of replacing ZnO with TCE on piglet intestinal morphology (n = 6). (**a**) Villus height; (**b**) Crypt depth; (**c**) Villus height:Crypt depth. Bars sharing different letter notations (a, b) are significantly different from each other (*p* < 0.05).

Compared to the control group, TCE increased ileal VH (*p* = 0.033) and reduced duodenal CD (*p* = 0.013). ZnO decreased jejunal CD (*p* = 0.000) and increased both duodenal (*p* = 0.042) and jejunal VH:CD (*p* = 0.000, Figure 5).

### 3.4. Effects of Replacing ZnO with TCE on Piglet Cecal Microbiota Composition

**Figure 6:**
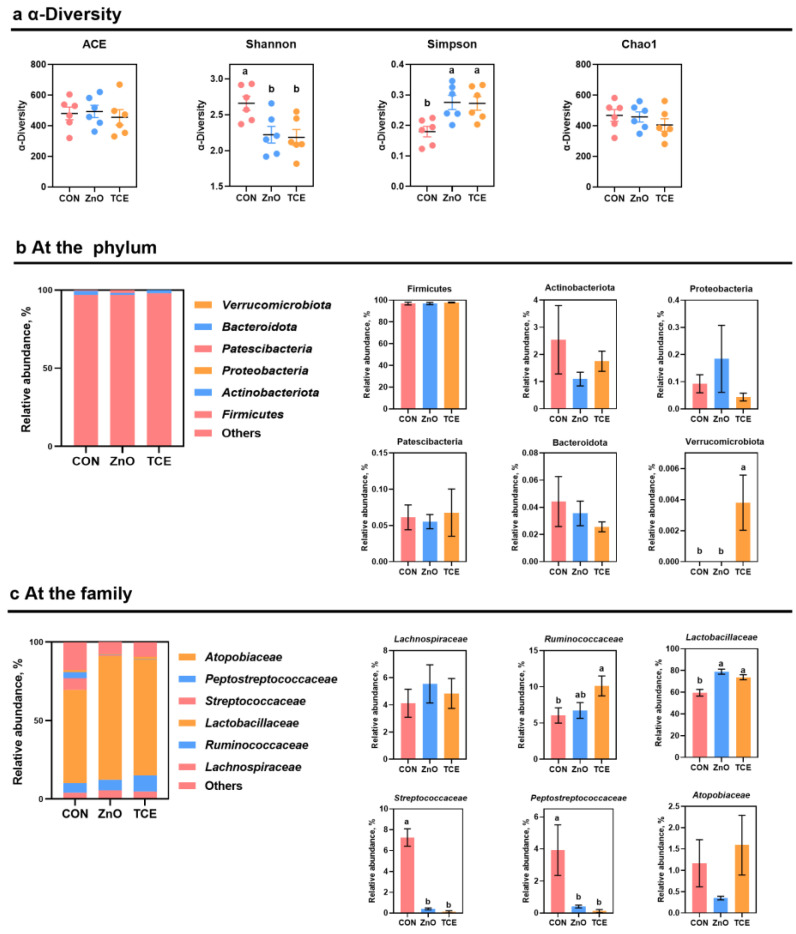
Effects of replacing ZnO with TCE on piglet cecal microbiota composition (n = 6). (**a**) α-Diversity; (**b**) at the phylum; (**c**) at the family. Bars sharing different letter notations (a, b) are significantly different from each other (*p* < 0.05).

Four alpha-diversity indexes (ACE, Chao, Simpson, and Shannon) were analyzed. Compared with CON group, the lower Shannon indexes (*p* = 0.039) and higher Simpson indexes (*p* = 0.022) were presented in the ZnO and TCE groups. However, no difference in the estimators (ACE and Chao) in results was observed among the three dietary treatments (*p* > 0.05; Figure 6a). At the phylum level, Firmicutes, Actinobacteriota, Proteobacteria, Patescibacteria, Bacteroidota, and Verrucomicrobiota were the top six phyla in the cecal microbiota. As shown in Figure 6b, the TCE group showed an increase in Verrucomicrobiota (*p* = 0.002). At the family level, *Lactobacillaceae*, *Ruminococcaceae*, *Lachnospiraceae*, *Streptococcaceae*, *Atopobiaceae,* and *peptostreptococcaceae* were the top six in the cecal microbiota (Figure 6c). Higher abundances of *Lactobacillaceae* (*p* = 0.000), and lower levels of *Streptococcaceae* (*p* = 0.000) and *Peptostreptococcaceae* (*p* = 0.035), in cecal contents were in the pigs fed the ZnO and TCE diets compared with CON pigs. Furthermore, compared with the CON group, TCE dietary treatment improved the abundance of *Ruminococcaceae* (*p* = 0.040). Additionally, administering ZnO or TCE in diets of pigs did not alter the *Lachnospiraceae* and *Atopobiaceae* compared to the CON group (*p* > 0.05).

### 3.5. Effects of Different Doses of TCE on Piglet Growth Performance, Diarrhea Rate, and Serum and Intestinal Mucosal Antioxidant Indices

**Figure 7:**
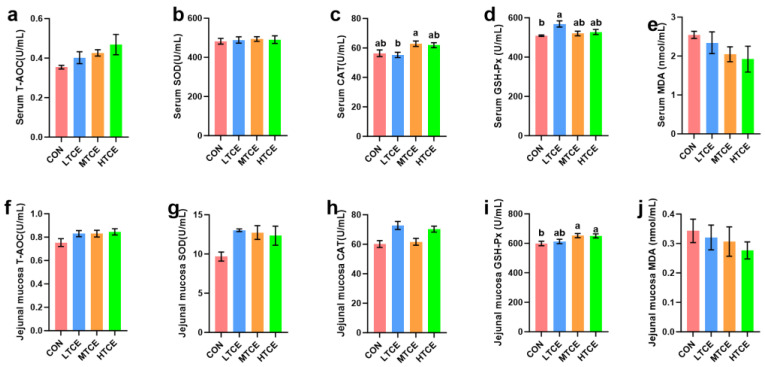
Effects of different doses of TCE on piglet serum and intestinal mucosal antioxidant indices (n = 6). (**a**) Serum T-AOC; (**b**) Serum SOD; (**c**) Serum CAT; (**d**) Serum GSH-px; (**e**) Serum MDA; (**f**) Jejunal mucosa T-AOC; (**g**) Jejunal mucosa SOD; (**h**) Jejunal mucosa CAT; (**i**) Jejunal mucosa GSH-px; (**j**) Jejunal mucosa MDA. Bars sharing the different letter notations (a, b) are significantly different from each other (*p* < 0.05).

As shown in Table 4, TCE increased ADG (*p* = 0.004), and both 1 g/kg and 4 g/kg of TCE reduced the F:G (*p* = 0.005) in piglets. As the dose of TCE increased, the diarrhea rate decreased (*p* = 0.014). Analysis of serum and intestinal related antioxidant indices indicated that LTCE group reduced serum CAT activity compared with the MTCE group (*p* = 0.021). Furthermore, compared with the CON group, the LTCE group increased GSH-Px activity in serum (*p* = 0.016), while the MTCE and HTCE groups increased GSH-Px activity in jejunal mucosa (*p* = 0.39, Figure 7).

### 3.6. Effects of Different Doses of TCE on Piglet Immunity

**Figure 8:**
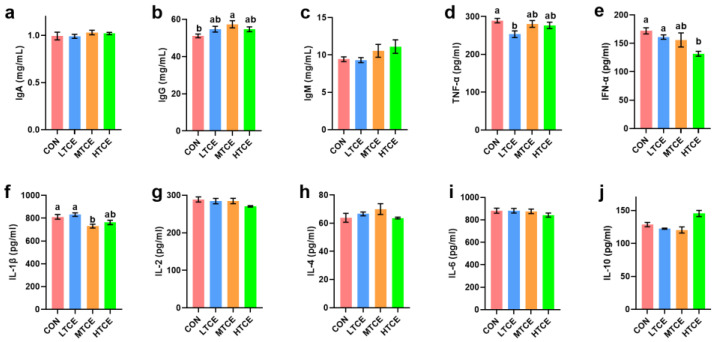
Effects of different doses of TCE on piglet serum inflammatory cytokines (n = 6). (**a**) IgA; (**b**) IgG; (**c**) IgM; (**d**) TNF-α; (**e**) INF-α; (**f**) IL-1β; (**g**) IL-2; (**h**) IL-4; (**i**) IL-6; (**j**) IL-10. Bars sharing different letter notations (a, b) are significantly different from each other (*p* < 0.05).

In serum, compared with the CON group, the MTCE group increased IgG levels (*p* = 0.047) and reduced IL-1β (*p* = 0.003), while the LTCE group reduced TNF-α (*p* = 0.043), and the HTCE group increased IFN-α (*p* = 0.007) concentrations (Figure 8).

**Figure 9:**
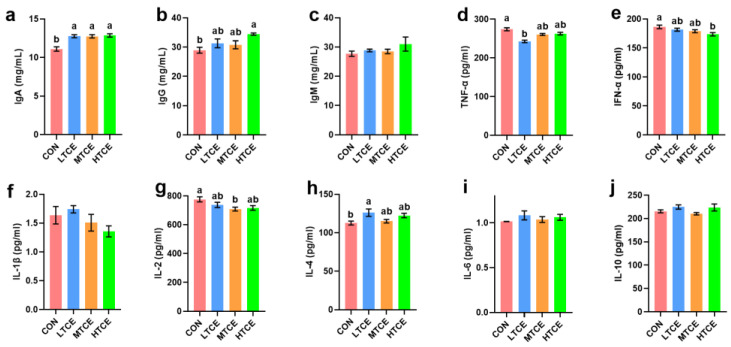
Effects of different doses of TCE on piglet jejunal mucosa inflammatory cytokines (n = 6). (**a**) IgA; (**b**) IgG; (**c**) IgM; (**d**) TNF-α; (**e**) IFN-α; (**f**) IL-1β; (**g**) IL-2; (**h**) IL-4; (**i**) IL-6; (**j**) IL-10. Bars sharing different letter notations (a, b) are significantly different from each other (*p* < 0.05).

Compared to the CON diet, TCE with different concentrations of supplementation increased IgA level in jejunal mucosa (*p* = 0.000), while TCE with middle-dose supplementation decreased the IL-2 level in jejunal mucosa (*p* = 0.039). Additionally, compared to CON pigs, pigs in the HTCE group had higher IgG (*p* = 0.039) and lower IFN-α (*p* = 0.030) levels in jejunal mucosa. Moreover, compared to the control group, the intestinal mucosal TNF-α (*p* = 0.000) was decreased and IL-4 (*p* = 0.030) was increased in LTCE group (Figure 9).

**Figure 10:**
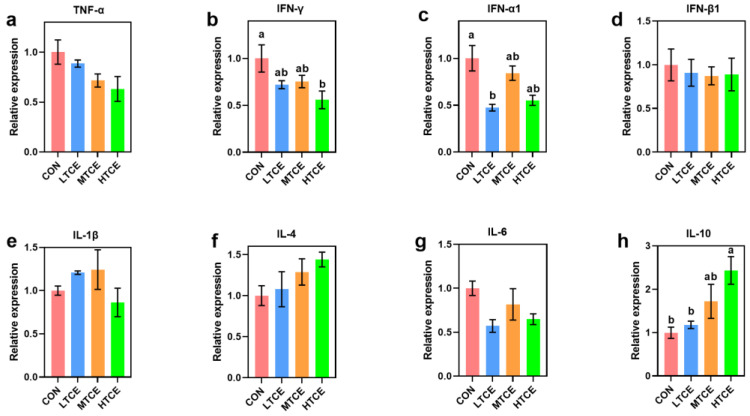
Effects of different doses of TCE on piglets relative to expression of inflammatory cytokines in the jejunal mucosa (n = 6). (**a**) TNF-α; (**b**) INF-γ; (**c**) IFN-α1; (**d**) IFN-β1; (**e**) IL-1β; (**f**) IL-4; (**g**) IL-6; (**h**) IL-10. Bars sharing different letter notations (a, b) are significantly different from each other (*p* < 0.05).

The results of relative expression for inflammatory cytokines in the jejunal mucosa were presented in Figure 10. Compared to the CON group, high doses of TCE reduced IFN-γ expression (*p* = 0.029), and low doses of TCE decreased IFN-α1 expression (*p* = 0.001). Compared to the CON and LTCE groups, IL-10 expression in the HTCE group was increased (*p* = 0.004).

### 3.7. Effects of Different Doses of TCE on Piglet Intestinal Morphology

**Figure 11:**
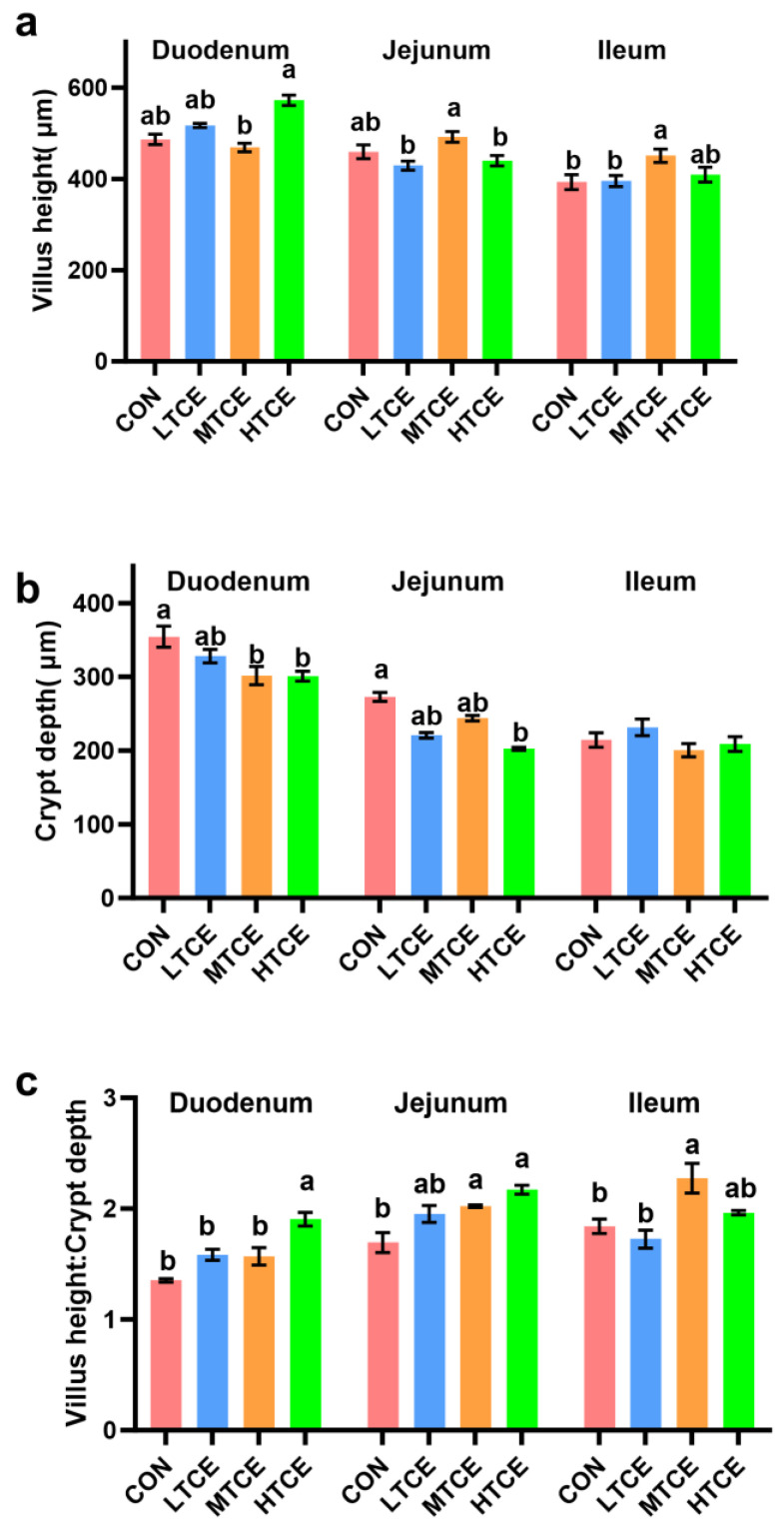
Effects of different doses of TCE on piglet intestinal morphology (n = 6). (**a**) Villus height; (**b**) Crypt depth; (**c**) Villus height: Crypt depth. Bars sharing different letter notations (a, b) are significantly different from each other (*p* < 0.05).

Compared with the MTCE group, the HTCE group increased duodenal VH (*p* = 0.000), while the LTCE and HTCE groups decreased the jejunum VH (*p* = 0.008, Figure 11a). Compared with the CON and LTCE groups, the MTCE group increased ileum VH (*p* = 0.045) and ileum VH:CD (*p* = 0.001). Compared with the CON group, the MTCE and HTCE groups decreased the CD in duodenal (*p* = 0.007) and jejunum (*p* = 0.000), and the HTCE group decreased the jejunum CD (*p* = 0.000, Figure 11b). Additionally, compared with CON, LTCE, and MTCE groups, the HTCE group increased the duodenum VH:CD (*p* < 0.000, Figure 11c). Compared with the CON group, the MTCE and HTCE groups increased jejunum VH:CD (*p* < 0.001).

## 4. Discussion

Under the influence of weaning stress and various diseases, piglets experience increased stress intensity, decreased immunity, and intestinal damage, it and may even lead to severe diarrhea and weight loss [17,18]. Against the backdrop of antibiotic prohibition, limited use of ZnO, and an emphasis on healthy farming practices, the development and application of feed additives such as plant extracts and probiotics have garnered widespread attention [19,20]. TCE exhibits strong antioxidant properties as well as antibacterial and anticancer effects [21,22], showing potential in alleviating piglet weaning stress and improving productivity. Rustia et al. demonstrated that TCE increases the G:F in fattening pigs [23]. Furthermore, TCE inhibits pathogens such as Escherichia coli, *Salmonella, Shigella, Clostridium perfringens*, and *Staphylococcus aureus* [24,25]. Previous studies also indicate that TCE can enhance the immune function and antioxidant capacity of broiler chickens, thereby improving intestinal health and growth performance [14]. TCE can increase the relative abundance of beneficial bacteria such as *Bacteroides* in the gut while reducing the relative abundance of harmful bacteria like *Alistipes*, thus alleviating inflammation and improving growth performance [14,26]. These studies collectively indicate that TCE can modulate the gut microbiota, enhance immune function, and enhance antioxidant capacity, thereby improving intestinal health and growth performance in animals. However, the effects of TCE as a substitute for ZnO on piglets and its potential mechanisms was not studied. The present study first found that both TCE and ZnO supplementation reduce the piglet diarrhea rate and increase ADG, with the reduction in diarrhea rate showing a dose-dependent relationship with TCE supplementation. Furthermore, 1 g/kg and 4 g/kg TCE reduce the F:G in piglets, suggesting that TCE may substitute for ZnO in controlling diarrhea and promoting piglet growth. Similarly, Xu et al., (2023) [27] demonstrated that 0.15% tannin could improve growth performance, digestibility, and intestinal function of weaned piglets; this positive effect may be associated with various biological functions of tannic acid, such as its astringency or anti-inflammatory effect, which has good potential to improve diarrhea and intestinal health of animals.

TCE exhibits strong antioxidant capacity, primarily attributed to polyphenolic compounds, polysaccharides, certain active proteins, and phenolic acid compounds [28,29]. Saha et al., (2016) [30] found that polyphenols contained in TCE have a phenolic hydroxyl group structure, which has the ability to scour free radicals. Furthermore, TCE can reduce serum MDA levels and increase GSH-px levels in diabetic patients, thereby relieving oxidative stress [10]. Moreover, TCE can reduce reactive oxygen species (ROS) levels, and enhance the activities of SOD, CAT, and GSH, thereby mitigating oxidative damage [26,31,32]. GSH-Px is one of the main antioxidant enzymes in animals, protecting cells from oxidative damage and reducing oxidative stress [13]. Our study found that both TCE and ZnO supplementation increase serum T-AOC. Furthermore, 2 or 4 g/kg TCE can increase duodenal mucosal GSH-Px activity, indicating that 2 or 4 g/kg TCE can substitute for ZnO to enhance piglet antioxidant capacity.

Plant extracts such as TCE can enhance animal immunity, thereby aiding animals in achieving better growth performance and health [33]. TCE can reduce colonic inflammation in mice with colitis [26]. TC exhibits a dose-dependent reduction in serum inflammatory markers in diabetic patients [10]. White lupin (another plant extract) in TCE reduces intestinal inflammation factors such as IFN-γ and TNF-α in mice infected with Salmonella, thereby enhancing mouse immunity [34]. Gallic acid and ellagic acid in TCE can inhibit the production of NO and IL-6 in LPS-induced RAW264.7 cells, exhibiting anti-inflammatory effects [35]. Hydrolyzable tannins in TCE can reduce serum levels of TNF-α, IL-1β, and IL-6 in a dose-dependent manner [36]. IgA is produced by plasma cells differentiated from B cells and reflects the strength of humoral immunity [37]. IL-10 is an anti-inflammatory cytokine with anti-inflammatory effects [38]. IFNs were initially considered substances that “interfere” with virus replication in vitro and are crucial for the immune response to most tested viruses [39]. In our study, different doses of TCE increased intestinal IgA levels and intestinal IL-10 expression, while serum and intestinal mucosal IFN-α levels decreased with increasing doses, indicating that TCE can enhance piglet immune function.

Intestinal morphology and development are closely related to an animal’s ability to absorb nutrients and serve as the first line of defense against harmful substances, making them primary indicators of intestinal health [40]. Studies have found that TCE alleviates pathological damage in mouse intestines, improving the VH:CD [34]. In this study, TCE reduced the duodenal CD and increased ileal VH, with increasing doses leading to an increase in the VHCD in both the duodenum and jejunum. This suggests that TCE can improve the intestinal morphology of piglets. In broiler chicken, TCE-containing diets for broilers resulted in a higher villus height and a higher ratio of villus height to crypt depth [40]. Thus, that research indicated that the integrity of intestinal morphology induced by TCE treatment may increase the beneficial effects on the gut and contribute to improved growth performance.

The gut is the primary site for microbial colonization, with its physiological and metabolic functions, such as nutrient absorption and immune protection, dependent on the gut microbiota [41,42]. Increasing evidence suggests that the gut microbiome may influence swine production. TCE exhibits antibacterial effects against organisms such as *Streptococcus mutans* and *Salmonella typhi* [24]. *Streptococcaceae* are marker microbes in the stools of patients with intestinal inflammation [43]. In addition, studies have shown that *Peptostreptococcaceae* are positively correlated with obesity and colorectal cancer [44,45]. *Lactobacillaceae*, a beneficial bacterium, can absorb plant-derived exosome-like nanoparticles to improve intestinal barrier functions, thereby alleviating intestinal diseases [46]. *Ruminococcaceae* form a multi-enzyme cellulolytic complex that can degrade plant cell wall polysaccharides, and TCE increases their presence in mouse feces [34].

We found that dietary supplementation of ZnO or TCE reduced the relative abundance of *Streptococcaceae* and *Peptostreptococcaceae* and increased the relative abundance of *Lactobacillaceae.* Furthermore, the TCE group showed an increase in *Ruminococcaceae* abundance. The anti-bacteria activity of TCE proved that the tannin component of TCE could prevent biofilm formation of pathogenic bacteria [47]. These results may support the dietary inclusion of TCE as an alternative to ZnO for increasing the quantity of beneficial microbes and reducing the number of harmful microbes in the piglet cecum, and inflammation-associated microbes, thereby regulating intestinal health.

## 5. Conclusions

In summary, dietary TCE was firstly presented as it can replace ZnO to improve piglet intestinal morphology, enhance antioxidant capacity, and boost immune function, as well as modulate the gut microbiome, thereby protecting the intestinal mucosa and promoting piglet growth. Furthermore, the recommended dose of TCE in piglet feed is 4 g/kg, positioning TCE as a potential feed additive.

## Figures and Tables

**Table 1:** Ingredients and nutrient levels of basal diets (%, as-fed basis).

Ingredients	CON	ZnO	LTCE	MTCE	HTCE
Corn (crude protein, 7.8%)	55.00	54.60	54.75	54.60	54.20
Full-fat soybean powder (crude protein, 35%)	10.00	10.00	10.00	10.00	10.00
Soybean meal (crude protein, 43%)	19.00	19.10	19.10	19.10	19.20
Whey powder	6.15	6.15	6.15	6.15	6.15
Fish meal (crude protein, 65%)	5.00	5.00	5.00	5.00	5.00
Soybean oil	1.50	1.60	1.55	1.60	1.70
L-Lysine·HCl (lysine, 78%)	0.48	0.48	0.48	0.48	0.48
DL-Methionine (methionine, 98%)	0.10	0.10	0.10	0.10	0.10
L-Threonine (threonine, 98%)	0.05	0.05	0.05	0.05	0.05
L-Tryptophan (tryptophan, 98%)	0.02	0.02	0.02	0.02	0.02
Dicalcium phosphate	0.90	0.90	0.90	0.90	0.90
Limestone	0.50	0.50	0.50	0.50	0.50
Sodium chloride	0.30	0.30	0.30	0.30	0.30
Compound vitamin and mineral Premix ^1^	1.00	1.00	1.00	1.00	1.00
Zinc oxide		0.2			
TCE			0.1	0.2	0.4
Total	100	100	100	100	100
Nutrient level ^2^					
Dry matter	86.78	86.82	86.80	86.82	86.86
Digestible energy (MJ/kg)	14.29	14.29	14.29	14.29	14.28
Crude protein	20.09	20.10	20.11	20.10	20.11
Lysine	1.49	1.49	1.49	1.49	1.49
Methionine	0.45	0.45	0.45	0.45	0.45
Methionine + cysteine	0.78	0.78	0.78	0.78	0.78
Threonine	0.82	0.82	0.82	0.82	0.82
Tryptophan	0.25	0.25	0.25	0.25	0.25
Calcium	0.74	0.74	0.74	0.74	0.74
Total phosphorus	0.64	0.64	0.64	0.64	0.64

^1^ The premix provide the following (per kg of the diet): vitamin A, 12,000 IU; vitamin D, 2500 IU; vitamin E, 30 IU; vitamin B_12_, 12 μg; vitamin K_3_, 3 mg; pantothenic acid, 15 mg; niacin, 40 mg; choline chloride, 400 mg; Mn (MnSO_4_·H_2_O), 40 mg; Zn (ZnSO_4_·H_2_O), 100 mg; Fe (FeSO_4_·H_2_O), 90 mg; Cu (CuSO_4_·5H_2_O), 8.8 mg; I (KI), 0.35 mg; Se (Na_2_SeO_3_), 0.3 mg. ^2^ The nutrient levels were calculated values.

**Table 2:** Primer sequences for real-time PCR.

Gene	Primers Sequences	Size (bp)	Accession No.
TNF-α	F: CCACGCTCTTCTGCCTACTGC	132	NM_214022.1
	R: CGACGGGCTTATCTGAGGTTTG		
IFN-γ	F: CCATTCAAAGGAGCATGGAT	146	NM_213948.1
	R: GAGTTCACTGATGGCTTTGC		
IFN-α1	F: GACTCCATCCTGGCTGTG	103	M28623
	R: TGACTTCTGCCCTGACGA		
IFN-β1	F: CAACAAAGGAGCAGCAAT	111	S41178
	R: CCTCAGGGACCTCAAAGT		
IL-1β	F: ATGCTGAAGGCTCTCCACCTC	89	NM_214055.1
	R: TTGTTGCTATCATCTCCTTGCAC		
IL-4	F: CAACCCTGGTCTGCTTACTG	173	NM_214123.1
	R: CTTCTCCGTCGTGTTCTCTG		
IL-6	F: CTTCAGTCCAGTCGCCTTCTCC	96	NM_001252429.1
	R: GCATCACCTTTGGCATCTTCTT		
IL-10	F: GGGCTATTTGTCCTGACTGC	105	NM_214041.1
	R: GGATTCTTCATCGGCTTCT		

**Table 3:** Effects of replacing ZnO with TCE on piglet growth performance and diarrhea rate.

Items	CON	ZnO	TCE	*p*-Value
Initial weight, kg	7.49 ± 0.34	7.39 ± 0.20	7.42 ± 0.22	0.962
Final weight, kg	18.60 ± 0.52	19.67 ± 0.57	19.81 ± 0.42	0.213
ADG, g/day	395.24 ± 9.29 ^b^	438.69 ± 12.22 ^a^	442.41 ± 10.96 ^a^	0.014
ADFI, g/day	747.03 ± 5.06	772.05 ± 4.42	769.71 ± 15.17	0.159
F:G (feed/gain, g/g)	1.85 ± 0.03	1.77 ± 0.04	1.74 ± 0.03	0.097
Diarrhea rate, %	5.50 ± 0.42 ^a^	2.08 ± 0.75 ^b^	3.32 ± 0.42 ^b^	0.002

Data are shown as the mean ± SEM. ^a,b^ Within a row, different superscripts refer to significant difference (*p* < 0.05). ADG: average daily gain; ADFI: average daily feed intake; F:G: feed-to-gain ratio.

**Table 4:** Effects of different doses of TCE on piglet growth performance and diarrhea rate.

Items	CON	LTCE	MTCE	HTCE	*p*-Value
Initial weight, kg	7.49 ± 0.34	7.38 ± 0.6	7.42 ± 0.22	7.39 ± 0.20	0.991
Final weight, kg	18.60 ± 0.52	20.03 ± 0.60	19.81 ± 0.42	19.96 ± 0.49	0.192
ADG, g/day	395.24 ± 9.29 ^b^	448.84 ± 10.31 ^a^	442.41 ± 10.96 ^a^	451.79 ± 11.69 ^a^	0.004
ADFI, g/day	747.03 ± 5.06	769.71 ± 15.17	769.71 ± 15.17	763.57 ± 11.67	0.497
F:G (feed/gain, g/g)	1.85 ± 0.03 ^a^	1.68 ± 0.02 ^b^	1.74 ± 0.03 ^ab^	1.70 ± 0.04 ^b^	0.005
Diarrhea rate, %	5.50 ± 0.42 ^a^	3.42 ± 0.58 ^ab^	3.32 ± 0.42 ^b^	2.99 ± 0.69 ^b^	0.014

Data are shown as the mean ± SEM. ^a,b^ Within a row, different superscripts indicate significant difference (*p* < 0.05). ADG: average daily gain; ADFI: average daily feed intake; F:G: feed to gain ratio.

## Data Availability

Dataset available on request from the authors.
